# Tweaking the Electronic and Optical Properties of α-MoO_3_ by Sulphur and Selenium Doping – a Density Functional Theory Study

**DOI:** 10.1038/s41598-018-28522-7

**Published:** 2018-07-04

**Authors:** Sateesh Bandaru, Govindarajan Saranya, Niall J. English, Chiyung Yam, Mingyang Chen

**Affiliations:** 1 Beijing Computational Science Research Center, Beijing, 100084 China; 2 School of Chemical and Bioprocess Engineering, University College Dublin, Belfield, Dublin 4, Ireland

## Abstract

First-principles calculations were carried out to understand how anionic isovalent-atom doping affects the electronic structures and optical properties of α-MoO_3_. The effects of the sulphur and selenium doping at the three unique oxygen sites (O_t_, O_a_, and O_t_) of α-MoO_3_ were examined. We found that the valence p orbitals of Sulphur/Selenium dopant atoms give rise to impurity bands above the valence band maximum in the band structure of α-MoO_3_. The number of impurity bands in the doped material depends on the specific doping sites and the local chemical environment of the dopants in MoO_3_. The impurity bands give rise to the enhanced optical absorptions of the S- and Se-doped MoO_3_ in the visible and infrared regions. At low local doping concentration, the effects of the dopant sites on the electronic structure of the material are additive, so increasing the doping concentration will enhance the optical absorption properties of the material in the visible and infrared regions. Further increasing the doping concentration will result in a larger gap between the maximum edge of impurity bands and the conduction band minimum, and will undermine the optical absorption in the visible and infrared region. Such effects are caused by the local geometry change at the high local doping concentration with the dopants displaced from the original O sites, so the resulting impurity bands are no long the superpositions of the impurity bands of each individual on-site dopant atom. Switching from S-doping to Se-doping decreases the gap between the maximum edge of the impurity bands and conduction band minimum, and leads to the optical absorption edge red-shifting further into the visible and infrared regions.

## Introduction

Molybdenum trioxide (MoO_3_) has garnered much research attention recently due to this material offering promising applications, coupled with its non-toxic nature, low cost and outstanding catalytic properties^1–7^. Further, MoO_3_ is found to be one of the most important metal oxides used as the electron-injection layers and the electrode material in the fast-growing field of photovoltaics and solar-cell devices. Amongst the three different polymorphic phases of MoO_3_, α-MoO_3_ with the orthorhombic layered crystal structure is thermodynamically most stable. α-MoO_3_ is an n-type semiconductor with a layered crystal structure with a wide band gap of 3.2 eV^8^. Due to its high band gap, α-MoO_3_ is not optimal to be used as photocatalyst for solar-energy applications directly. In order to utilize α-MoO_3_ effectively as a photocatalyst, we need to modify its structure to reduce the band gap for the improved efficiency in harness the solar energy of which the major component is from the visible region. So far, significant progress has been made in the fabrication and modification of α-MoO_3_-based materials and devices, and the performance and reliability of the material has been greatly improved. Nano-sized MoO_3_, such as nanoporous, nanobelts and nanorods, has drawn substantial attention and been utilized in various applications, such as electrochromic/photochromic devices^9^, pseudocapacitive charge storage^10^, supercapacitors^11,12^, gas sensors^13,14^, lithium-ion batteries^15^ and effective heterogeneous catalysts^16^. Kumar *et al*.^10^ engineered the surface of MoO_3_ nanobelts for electrochemical cell applications and reported that their specific capacitance is enhanced with relatively high stability. Hamwi *et al*.^17^ studied p-type doping efficiency of MoO_3_ in organic hole-transport materials and White *et al*.^18^ studied the interface structure of MoO_3_ and organic semiconductors for organic electronic device applications. Experimentally, Qin *et al*. studied^19^ the sulfur-doped molybdenum oxide (S-MoO_3_) material used as anode interface layer and their study shows that doping of S atom to the MoO_3_ can effectively modify anode interface layer to improve the hole-transport properties of MoO_3_.

The electronic, optical and catalytic properties of MoO_3_ are related to various factors including the structures, defects, and impurities. Earlier, White *et al*.^18^ examined the effects of surface structure, oxygen vacancy, and hydrogen adsorption on the catalytic properties on MoO_3_ using the DFT+U method and rationalized its reaction mechanisms^20^. The recent work by Agarwal *et al*. explored the catalytic applications of the bulk MoO_3_ and two-dimensional MoO_3_^21^. Yang *et al*.^22^ investigated MoO_3_-TiO_2_ nanotube arrays by electrochemical anodization and reported an improved efficiency for the separation of photoinduced carrier pairs and an enhanced water-splitting performance. Yu *et al*.^23^ reported MoO_3_-TiO_2_ composite nanorods films that exhibited improved electrochromic performance. Recently, Chen *et al*.^24^ synthesized MoO_3_ nanobelts and the proposed that the impurity band (IB) in the gap is due to the oxygen vacancies. Among the various factors that have substantial influences on the electronic, optical and catalytic properties of MoO_3_, the impurity that can be introduced facilely via the doping treatment can be used as a conventional way to reduce the band gap of α-MoO_3_ for solar energy related applications.

Sulphur and selenium are group VIA elements that are isovalent to oxygen, but have higher valence p orbital energies than O (the first ionization energies of S and Se are 10.36^25^ and 9.75 eV^26^ respectively, lower than the first ionization energy of O 13.62 eV)^27^. Therefore, substituting S or Se for O may lead to substantial changes to the optical properties of α-MoO_3_. The effects of S-doping to WO_3_ (both W and Mo are group VIB transition metals) have been previously examined by Wang *et al*.^28^. Their study shows the S dopants can introduce impurity bands inside the band gap, and the resulting HOMO-LUMO gap (from the maximum edge of impurity bands to conduction band minimum) is smaller than the band gap of the pure material, which facilitates the absorption of visible light. However, to the best of our knowledge, there has not been a systematic first-principles study on how anionic (S and Se) doping at different doping sites and at different concentration affects the electronic structure and optical behaviour of α-MoO_3_. In order to understand how such doping processes affect the properties of α-MoO_3_, sulphur and selenium atoms were doped into the 3 unique oxygen sites of α-MoO_3_ at different local doping concentrations modelled using the first-principles calculations at the density functional theory (DFT) level. Our study is an important step forward to understand the effects of the ionic doping on α-MoO_3_, and to learn to tweak the electronic and optical properties of α-MoO_3_ by controlling the simple factors such as the doping concentration.

## Computational Details

The first-principles calculations were carried out using Perdew–Burke–Ernzerhof (PBE) exchange-correlation functional within generalized gradient approximation (GGA), implemented in the Vienna *ab initio* simulation package (VASP) code^29,30^. The projected augmented wave (PAW) method^31^ with a plane-wave basis set was used. All of the structures were fully relaxed without any symmetric constraints. The energy cutoff is set to 550 eV and the convergence criteria for energy and force are set to 10^−5^ eV and 0.01 eV/Å, respectively. In order to better take the effect of the on-site Coulomb repulsion of Mo 3d electrons into account, the exchange correlation energy was treated by Perdew, Burke, and Ernzerhof (PBE)^32^ functional, in conjunction with the PBE+U (U_eff_ = 8.6 eV for the Mo d electrons) approach of Dudarev *et al*.^33,34^. First, we tested different U_eff_ values ranging from the 3.0 to 8.6 eV for the calculation of the raw MoO_3_ material. The U_eff_ value that predicts band gap in the best agreement with the experimental values was then used in the calculations of the doped MoO_3_ systems. For the band structure calculations, the integration over the first Brillouin zone was performed using a 4 × 4 × 3 Monkhorst-Pack k-point grid^35^. In our first-principles studies, the lattice constants of the supercells were kept constant during the doping process, so that the change in electronic stucture is caused the dopants rather than by resizing the super cell.

**Figure 1:**
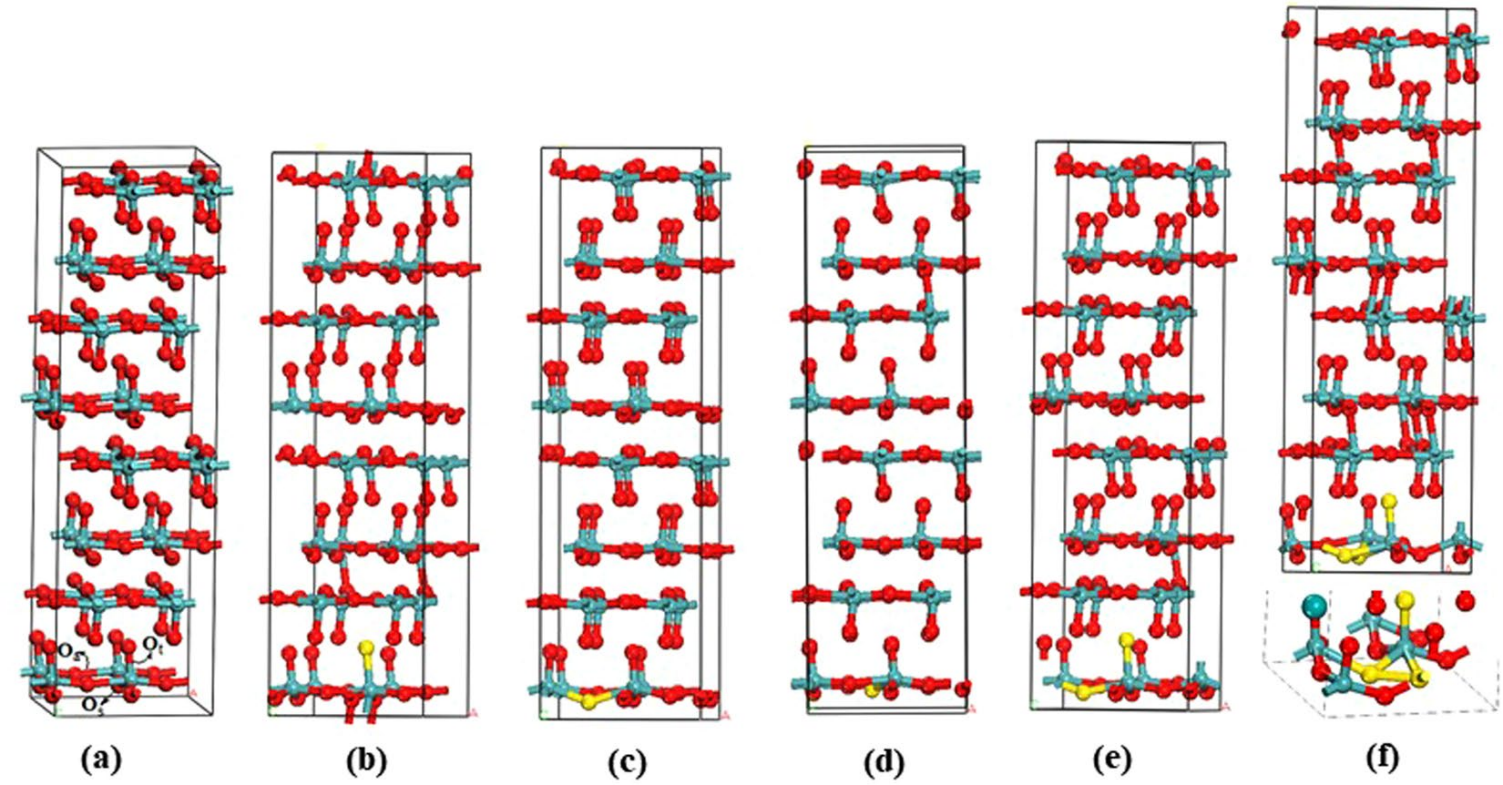
Constructed supercell for **(a)** the bulk α-MoO_3_(2 × 2 × 2); **(b**–**d)** the *mono-*S doped MoO_2.97_(S_t_)_0.03_, MoO_2.97_(S_a_)_0.03_, and MoO_2.97_(S_s_)_0.03_ respectively; **(e)** MoO_2.94_(S_t_S_a_)_0.03_ is the *bi-*S and (**f**) MoO_2.91_(S_t_S_a_S_s_)_0.03_ is the *tri*-S doped systems in the MoO_3_ (2 × 2 × 2) supercell.

We have used a 2 × 2 × 2 MoO_3_ host supercell that consists of 32 Mo atoms and 96 O atoms (Fig. 1) to simulate the anionic doped MoO_3-x_S_x_ and MoO_3-x_Se_x_ with different sulphur and selenium concentrations (indicated by different values of x). This doping process can be achieved by substituting oxygen atoms of the super cell with the dopant atoms (S/Se). Substituting one, two and three oxygen atoms of the supercell yields x = 0.03, 0.06 and 0.09 respectively. Bulk α-MoO_3_ has three distinct types of oxygen atoms: terminal (O_t_), asymmetrical (O_a_), and symmetrical (O_s_) oxygen atoms (Fig. 1a)^20^. The O_a_ is 2-fold by forming one long (2.20 Å) and one short (1.78 Å) bonds with two Mo atoms in the same layer. The O_s_ is 3-fold as it forms two equal intralayer Mo-O bonds (1.95 Å) and one longer interlayer Mo-O bond (2.38 Å). The O_t_ forms one Mo=O bond with a single Mo atom, for which the bond distance (1.68 Å) is the shortest in the system.

Replacing one of the three oxygen site variants (O_t_, O_s_ and O_a_) of the supercell with one S atom leads to three unique *mono*-atomic S-doped structures, namely MoO_2.97_(S_t_)_0.03,_ MoO_2.97_(S_a_)_0.03_, and MoO_2.97_(S_s_)_0.03_, with a Mo_32_O_95_S formula for the supercell. The *bi*-S doped structure was constructed by replacing an O_t_ and an O_a_ about the same Mo in the supercell, and the resulting structure is denoted as MoO_2.94_(S_t_S_a_)_0.03_ with a Mo_32_O_94_S_2_ formula for the supercell. The *tri*-S doped structure was constructed by replacing an O_t_, and an O_a_, and an O_s_ about the same Mo in the supercell, denoted as MoO_2.91_(S_t_S_a_S_s_)_0.03_ with a Mo_32_O_93_S_3_ formula. Similar construction procedures were applied to generate the Se-doped structures (Fig. S1).

In addition, to examine the doping effects of uniform doping (*i.e*. all the dopant atoms substituting for the same type of O site) at higher concentration, in contrast to the *bi*- and *tri*- S doped at the same Mo-site, we also explored the *bi*-S and *tri*-S doped structures where single-type O sites about multiple Mo atoms in the supercell are replaced by the dopants. The *bi-S doped structure* was constructed by replacing two O_t_ atoms that are coordinated to two different Mo’s in the supercell, denoted as MoO_2.94_(S_t_)_0.06_ with a Mo_32_O_94_S_2_ formula. The *tri*-S doped structure was constructed by replacing three O_t_ atoms (about three Mo atoms) in the supercell, denoted as MoO_2.91_(S_t_)_0.09_ with a Mo_32_O_93_S_3_ formula. These corresponding relaxed structures are shown in Fig. S2(a,b) in the supplementary information (SI), and similar construction for Se-doped structures with higher dopant concentrations are shown in Fig. S2(c,d) in the SI.

The optical absorption spectra of pure and S/Se doped systems were calculated based on the dielectric function. The imaginary part ε_2_(ω) of the dielectric function can be calculated from the momentum matrix elements between the occupied and unoccupied wave functions with appropriate selection rules^36^. The real part ε_1_(ω) of the dielectric function was obtained by the Kramer–Kronigs relations. The optical absorption coefficient *α*(*ω*) can be computed using following formula

$$\alpha (\omega )=\sqrt{2}\omega {[\sqrt{{\varepsilon }_{1}^{2}(\omega )+{\varepsilon }_{2}^{2}(\omega )-{\varepsilon }_{1}(\omega )}]}^{\frac{1}{2}}$$

### Availability of materials and data

All data generated or analyzed during this study are included in this published article (and its Supplementary Information files).

## Results and Discussion

For the DFT + U calculations of α-MoO_3_, a scattering range of U_eff_ values from 1 to 8.6 eV have been used in the literature^21^. The value of U_eff_ is usually empirically determined, and it is near impossible to find a universal U_eff_ that is effective for all the different cases. In the current study, the U_eff_ value that predicts lattice parameters, Mo-O bond distances and band gap (E_g_) in the best agreement with the experimental data for the bulk α-MoO_3_ was chosen to be applied to the MoO_3-x_S_x_ and MoO_3-x_Se_x_ calculations. The calculated lattice parameters and band gap values at the PBE+U level with different U_eff_ values are given in Table S1 in the SI. From the Table S1, we clearly observed that at the PBE level essentially all of the tested U_eff_ values predict lattice parameters and Mo-O bond distances in good agreements with the experimental values^37^, *i.e*., within ~1% of the experimental values. Different U_eff_ values, however, result in different E_g_ prediction values, ranging from 1.55 to 2.58 eV (Table S1 and Fig. S3 in the SI). The closest E_g_ value to the experimental value (3.2 eV)^38,39^ is given by U_eff_ = 8.6 eV. The underestimation of E_g_ (by this study and previous studies)^40^ is mostly due to the inadequacy of the theory that DFT fails to accurately describe the relative energies of occupied and unoccupied electron energy levels. The present paper is focused to see how the anionic atom doping affects the observed band gaps and the presence of the impurity bands (gap states), for which the E_g_ shifts and the relative positions of the impurity bands in response to the different concentrations of the impurity anion are particularly interested. For the systems studied in the current study, the doping concentration is not high enough to affect the bulk electronic structure, and hence the doping effect on E_g_ is negligible (see below discussions). We also tested using different U_eff_, the calculated energy difference between the impurity band and valence band maximum for a doped MoO_3_ system (Table S2 in the SI) and found the impurity band–valence band maximum gap is essentially invariant to the choice of U_eff_. Hence, we have proceeded our calculations with U_eff_ = 8.6 eV.

**Figure 2:**
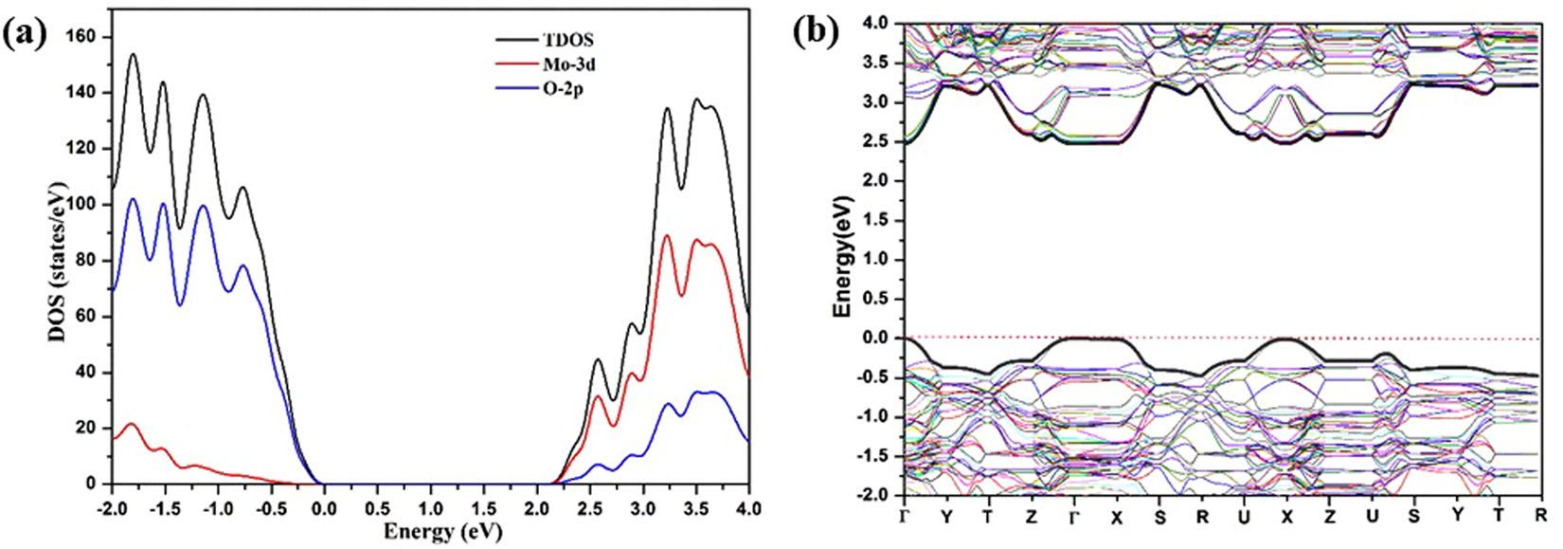
(**a**) DOS and (**b**) band structure for the bulk α-MoO_3_ (2 × 2 × 2) supercell. Zero energy is set to the energy level of the valence band maximum.

Before we discuss the S/Se- doping effects, initial calculations have been carried out on the bulk MoO_3_ (2 × 2 × 2) without any symmetry constrains (with the optimized structure shown in Fig. 1a); the calculated bond distances of MoO_3_ are 1.68 Å for Mo-O_t_, 1.77 Å and 2.21 Å for Mo-O_a_, and 1.95 Å for Mo-O_s_, respectively. These bond distances are in good agreement with the experimentally measured unit cell parameters (Table S1 in the SI). The predicted density of states (DOS) for the pure α-MoO_3_(2 × 2 × 2) indicates that the valence band edge of the material is dominated by O 2p states and conduction band edge is dominated by Mo 3d states (Fig. 2). The calculated band structure of α-MoO_3_ shows that the valence band maximum (VBM) and conduction band minimum (CBM) are at Γ, suggesting α-MoO_3_ is a direct band gap semiconductor. The band edges at X are comparable in energy to the edges at Γ. At U, the valence band edge is ~0.2 eV lower than the VBM and the conduction band edge is ~0.1 eV higher than the CBM. This is different from the prior prediction by Scanlon *et al*. using the PBE functional^41^. Their study suggested that the VBM is situated at U and the CBM is at Γ, which lead to a minimal indirect gap of 1.95 eV and a minimal direct gap of 2.76 eV.

### Doping sites favorability

The optimized geometries of the *mono-*S doped MoO_2.97_(S_t_)_0.03_, and MoO_2.97_(S_a_)_0.03_ and MoO_2.97_(S_s_)_0.03_ (Fig. 1(b,c)) structures are essentially similar to the pure α-MoO_3_. The Mo-S bonds, however, are longer than the corresponding Mo-O bonds because S has a large ionic radius than O. In the *mono*-S doped MoO_2.97_(S_t_)_0.03_ system, the dopant S_t_ forms one Mo-S_t_ bond with the surrounding environment, and the Mo-S_t_ bond distance is 2.073 Å. In MoO_2.97_(S_a_)_0.03_, S_a_ forms two unequal Mo-S bonds with two Mo atoms, and the corresponding distances are 2.333 and 2.166 Å (Table S3 in the SI). In MoO_2.97_(S_s_)_0.03_ S_s_ forms three equal Mo-S bonds with three Mo atoms with a distance of 2.297 Å. It is noted that all the Mo-S bonds are longer than their Mo-O counterparts, which is mostly due to S having the greater ionic radius than O. The bond length difference between the two Mo-S_a_ bonds (0.17 Å) of MoO_2.97_(S_a_)_0.03_ is found to be much smaller than the difference between the two Mo-O_a_ bonds (0.42 Å) of the pure material, indicating a substantial change in the local geometry upon the S_a_-doping treatment. For the *mono*-Se doped systems, Mo-Se_t_, Mo-Se_a_ and Mo-Se_s_ bond distances are 2.199, 2.445(2.270) and 2.405 Å, respectively (Table S4 in the SI). The stabilities and electronic structures of *mono-*S doped MoO_3_(2 × 2 × 2) structures with one S atom substituted for each type of oxygen site (O_t_, O_a_, and O_s_) were examined. Among the three *mono-*S substituted structures, MoO_2.97_(S_t_)_0.03_ (Fig. 1) is found to be more stable than the MoO_2.97_(S_a_)_0.03_ and MoO_2.97_(S_s_)_0.03_ (the corresponding relative energies are 0.0, 1.14 and 0.99 eV respectively). Similarly, and the results show that MoO_2.97_(Se_t_)_0.03_ (Fig. S1 in SI)) is more stable than MoO_2.97_(Se_a_)_0.03_ and MoO_2.97_(Se_s_)_0.03_ with the corresponding relative energies being 0.0, 1.63 and 1.29 eV respectively.

Formation energies of *mono*-S and Se doped α-MoO_3_(2 × 2 × 2) were computed using the first principle calculations. The formation energies (E_*form*_) for a doped system from a pure system can be calculated by using Eq. (1):

1 $${E}_{form}=\lfloor {E}_{(doped)}-({E}_{(pure)}-n{{\rm{\mu }}}_{O}+n{{\rm{\mu }}}_{S})\rfloor $$

where *E*_(*doped*)_ and *E*_(*pure*)_ are the total energies of doped and perfect α-MoO_3_(2 × 2 × 2) respectively, and μ_*O*_ and μ_*S*_ are the chemical potential of the O and S atoms respectively and *n* is the number of substituted atoms (n = 1 to 3) in the supercell. From the equation (1) the calculated *E*_*form*_ are 2.64 and 2.15 eV for the *mono-*S doped MoO_2.97_(S_t_)_0.03_ and the *mono*-Se doped MoO_2.97_(Se_t_)_0.03_ respectively (Table S5 in the SI). This suggests that the Se doping is more energetically preferable than the S-doping. We obtained the reaction energy for the solid S/Se doping reaction with α-MoO_3_ (to release O_2_) by correcting *E*_*form*_ with the experimental heats of formation data^42^ of gas-phase O, S, and Se, for which the details can be found in SI. The reaction energies for doping α-MoO_3_ with solid-phase S and Se are 2.95 and 1.92 eV respectively. Both doping reactions are found to be highly endothermic, and thus it might require high temperature for the doping reactions to occur.

### Site-dependent doping effects

**Figure 3:**
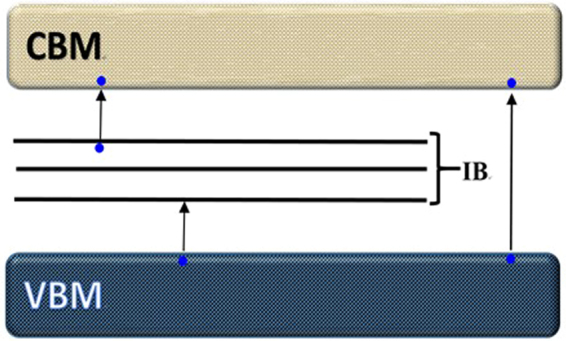
The impurity bands were observed in S and Se doped α-MoO_3_(2 × 2 × 2).

Previous experimental and theoretical studies have suggested that both the oxygen vacancies and dopants could give rise to the impurity bands (IBs, Fig. 3)^43,44^. We found that impurity bands (IBs, Fig. 3) situated above the VBM in the band structure of MoO_3_ upon the S- and Se- doping, even at low concentration. Similar IBs have been reported in a previous study on Sn-doped chalcopyrite’s^45^ by Yang *et al*. We also found the number of IBs of the doped structure is dependent on which oxygen site was doped into. Two IBs are observed in the band structure of MoO_2.97_(S_a_)_0.03_, whereas one IB is observed in the band structure of MoO_2.97_(S_t_)_0.03_ and MoO_2.97_(S_s_)_0.03_.

**Figure 4:**
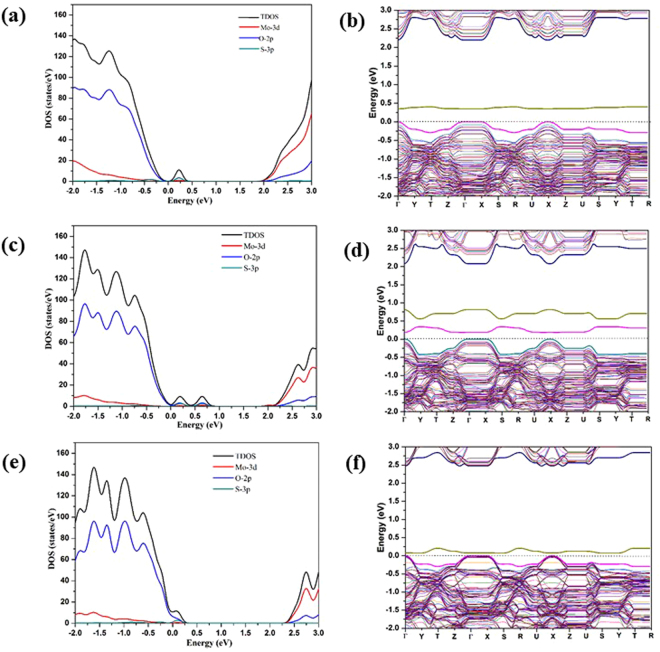
(**a**,**c**,**e**) The TDOS and the PDOS for MoO_2.97_(S_t_)_0.03,_ MoO_2.97_(S_a_)_0.03_, and MoO_2.97_(S_s_)_0.03_ respectively; (**b**,**d**,**f**) the band structures for MoO_2.97_(S_t_)_0.03,_ MoO_2.97_(S_a_)_0.03_, and MoO_2.97_(S_s_)_0.03_ respectively at the PBE+U level (U_eff_ = 8.6 eV) with zero energy set to the VBM of the pure α-MoO_3_.

The calculated DOS’s and band structures for the *mono*-S doped MoO_2.97_(S_t_)_0.03,_ MoO_2.97_(S_a_)_0.03_, and MoO_2.97_(S_s_)_0.03_, structures are shown in Fig. 4. The IBs for all of the *mono*-S doped structures are found close to the corresponding VBM. For the cases of MoO_2.97_(S_t_)_0.03_ and MoO_2.97_(S_s_)_0.03_, a single IB are observed at 0.26 and 0.1 eV above the VBM, respectively; whereas for the case of MoO_2.97_(S_a_)_0.03_, two IBs were observed at 0.24 eV and 0.75 eV above the VBM. The band structure diagram shows there are in fact two IBs above the VBM for MoO_2.97_(S_t_)_0.03_, but the lower energy one is only ~0.1 eV above the VBM of pure material at Γ, which is not obviously shown in DOS. From the DOS, we clearly observed that all of the impurity bands have contributions from the 3p orbitals of S atom which are largely mixed with the 2p orbitals of O atoms nearby. The energy levels of the IBs for the three *mono*-doped structures are different. The IB for the MoO_2.97_(S_s_)_0.03_ structure is ~0.1 eV above the VBM of the pure MoO_3_ structure, being the closest to the VBM. The IB for the MoO_2.97_(S_t_)_0.03_ and the lower energy IB for MoO_2.97_(S_a_)_0.03_ is about 0.4 eV above the VBM of the pure MoO_3_, whereas the higher energy IB for MoO_2.97_(S_a_)_0.03_ is ~0.7 eV above the VBM of the pure MoO_3_. We think that there might be a correlation between the coordination of the S dopant atom at different doping sites and the energy level of the IB. The S_t_ forms a double bond with Mo and S_a_ forms two single bonds with two Mo’s. In both cases, there is a lone pair of 3p electrons that has no significant interaction with the surrounding atoms, of which the energy levels are higher than the energy levels of the bonding orbitals. These lone pairs are most likely to be responsible for the higher-energy IB of MoO_2.97_(S_t_)_0.03_ and MoO_2.97_(S_a_)_0.03_. The higher-energy IB of MoO_2.97_(S_a_)_0.03_ is at higher energy level than the higher-energy IB of MoO_2.97_(S_t_)_0.03_, possibly because the destabilization effect of the excessively large Mo-O_a_-Mo angle (~154°). The lower-energy IB of MoO_2.97_(S_t_)_0.03_ is attributed to the Mo-S_t_ π orbital, which is almost buried by the valence band edge. The lower-energy IB of MoO_2.97_(S_a_)_0.03_ is attributed to one of the Mo-S_a_ σ orbital whose energy is raised due to the John-Teller distortion as a consequence of the two unequal Mo-S_a_ bonds. The S_s_ is coordinated to 3 Mo’s with two short intralayer Mo-S bonds and one long interlayer Mo-S bond (Table S3 in the SI). Clearly, the lone pair of S_s_ forms an interlayer donor-acceptor bond with a Mo of the adjacent layer, and the involved p-electrons have higher level than the p states that forms the two intralayer bonds but have slightly lower energy level than the lone pair states of the S_t_-doped systems. Therefore, there is only a single IB close in energy to the VBM for MoO_2.97_(S_s_)_0.03_.

According to the calculated PDOS for the bands attributed to the three S 3p orbitals (Fig. S4 in the SI) for the *mono-*S doped MoO_3_ structures, the average energy levels of the three S 3p orbitals with respect to the VBM of the pure α-MoO_3_ are: MoO_2.97_(S_t_)_0.03_ < MoO_2.97_(S_s_)_0.03_ < MoO_2.97_(S_a_)_0.03_, which is consistent with the stability ranking of the three *mono-*S doped structures. This can be expected by treating the electrons as non-interacting electrons and writing the total energy as the integral of the population-weighted valence band energies (*i. e*. the band energy).

From the band-structure calculations, the band gap of bulk α-MoO_3_(2 × 2 × 2) is 2.5 eV using PBE+U. For *mono*-S doped structures band gaps are ~2.2, ~2.1 and ~2.45 eV for the S_t_, S_a_ and S_s_ doping, respectively. The decreases in the band gaps with respect to the band gap of the pure MoO_3_ (calculated to be 2.6 eV) are mainly due to the emergences of the unoccupied gap states closely below CBM upon the doping, and also due to the distorted local geometries of the doped materials that shift the whole VBM towards lower energy, as indicated by the band structures of the doped materials (Fig. 4). Among the three doping positions, S doping at O_a_ position is most suitable for the reduction of band gap.

**Figure 5:**
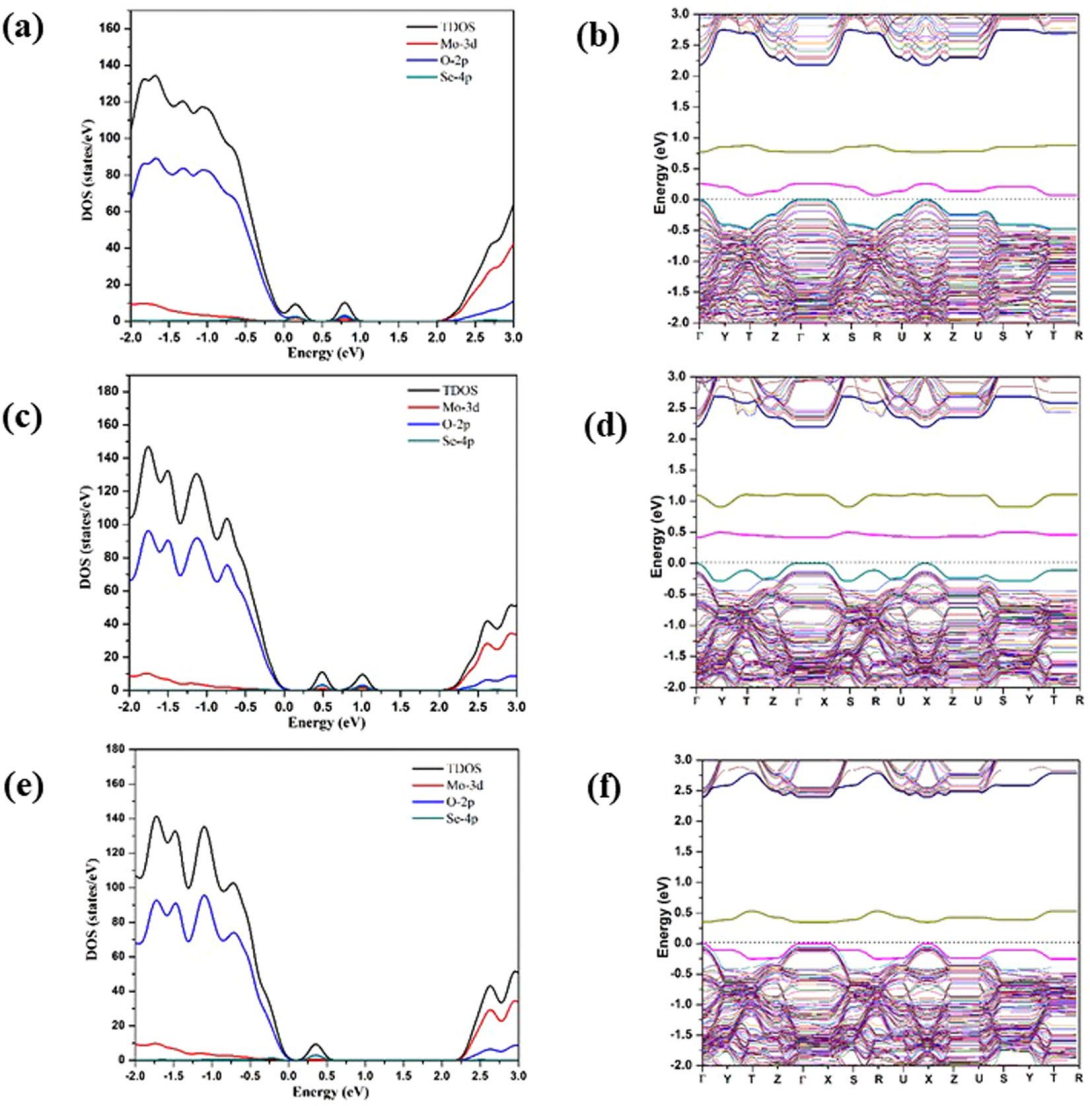
(**a**,**c**,**e**) The total density of states (TDOS) and the Mo 3d, O 2p and S 3p projected density of states (PDOS) for MoO_2.97_(Se_t_)_0.03_, MoO_2.97_(Se_a_)_0.03_ and MoO_2.97_(Se_s_)_0.03_; (**b**,**d**,**f**) the calculated band structures for MoO_2.97_(Se_t_)_0.03_, MoO_2.97_(Se_a_)_0.03_ and MoO_2.97_(Se_s_)_0.03_ at the PBE+U level (U_eff_ = 8.6 eV) with zero energy set to the VBM of the pure α-MoO_3_.

Similar IBs were also observed in the *mono*-Se doped MoO_3_ structures as shown in Fig. 5. Two IBs were observed for both MoO_2.97_(Se_t_)_0.03_ and MoO_2.97_(Se_a_)_0.03_ structures, whereas in the case of MoO_2.97_(Se_s_)_0.03_ only a single IB was observed in the band-gap area. For MoO_2.97_(Se_t_)_0.03_ the newly-introduced bands are ~0.25 and 0.75 eV above the VBM of the pure α-MoO_3_ (cf. Fig. 5(d)). For MoO_2.97_(Se_a_)_0.03_, the two IBs are ~0.5 eV and 1.1 eV above the VBM. The latter IB is almost located in middle of the VBM and CBM. Similar explanations as what we gave for the cases of the *mono*-S doped structures can be applied to explain the rises of the IBs for the *mono-*Se doped structures. The IBs that appear in the electronic structures of the *mono-*Se doped structures are found to be higher in energy than the IBs that appear for the *mono-*S doped structures, as the valence p states of Se are higher in energy than the valence p states of S. Comparing to the S *mono*-doped systems, the impurity band in MoO_2.97_(Se_s_)_0.03_ is slightly higher than that of in MoO_2.97_(S_s_)_0.03_. For MoO_2.97_(Se_s_)_0.03_ the single impurity band is found at 0.25 eV above to the VBM, whereas for MoO_2.97_(S_s_)_0.03_ the IB is 0.1 eV above the VBM. As for MoO_2.97_(Se_t_)_0.03_ and MoO_2.97_ (Se_a_)_0.03_, a slight reduction was observed in the band gap. Overall, for the *mono*- S and Se doped systems, similar kind of impurity bands were observed, but the position of IB bands are different in both cases. The energy gap between the highest energy IB and the CBM for a *mono*-Se doped system is therefore smaller than the gap for the corresponding *mono*-S doped system. This might result in different optical absorption behaviors for the two classes of doped MoO_3_ material in the visible and infrared ranges. The band gap of *mono*-Se doped systems are 2.24, 2.20 and 2.35 eV for the Se_t_, Se_a_ and Se_s_ doping, respectively, which are comparable to the band gaps of the corresponding *mono*-S doped systems.

### Tuning the doping effects with doping concentration

To explore the S and Se atoms doping about the same Mo-site, which is related to the practical situations where the dopant concentration is high and the local concentration (about a Mo) increases, we have carried out the geometry optimization calculations for the *bi*-S and *tri*-S doped systems, namely MoO_2.94_(S_t_S_a_)_0.03_ and MoO_2.91_(S_t_S_a_S_s_)_0.03_, and the resulting optimized structures are shown in Fig. 1(e,f). For the *tri*-S doped MoO_2.91_(S_t_S_a_S_s_)_0.03_ systems, we have all three variants of oxygens (O_t_O_a_O_s_) sites replaced about the same Mo atom by S atoms. Unlike the cases of the *mono*-S doped MoO_3_ structures and the *bi*- doped MoO_2.94_(S_t_S_a_)_0.03_, where all the S dopant atoms sit essentially at the original O sites, the S_s_ dopant in the optimized geometry of MoO_2.91_(S_t_S_a_S_s_)_0.03_ is found to be out of the position of O_s_ and form a S-S bond (2.045 Å) with S_a_. The S_s_ no longer form two equal intralayer Mo-S_s_ bonds with two Mo atoms; instead, the two intralayer Mo-S_s_ bond distances are found to be 2.352 and 2.479 Å in MoO_2.91_(S_t_S_a_S_s_)_0.03_. The interlayer Mo-S_s_ distance is found to be essentially the same as the interlayer Mo-S_s_ distance in the *mono*-S doped MoO_2.97_(S_s_)_0.03_, indicating that the displacement of S_s_ in MoO_2.91_(S_t_S_a_S_s_)_0.03_ is approximated confined in the xy-plane. The formation of the S_s_-S_a_ bond also affects the bonding between S_a_ and Mo’s. The bond distance of the two Mo-S_*a*_ bonds in MoO_2.91_(S_t_S_a_S_s_)_0.03_ are 2.391 and 2.357 Å, which are approximately equal.

**Figure 6:**
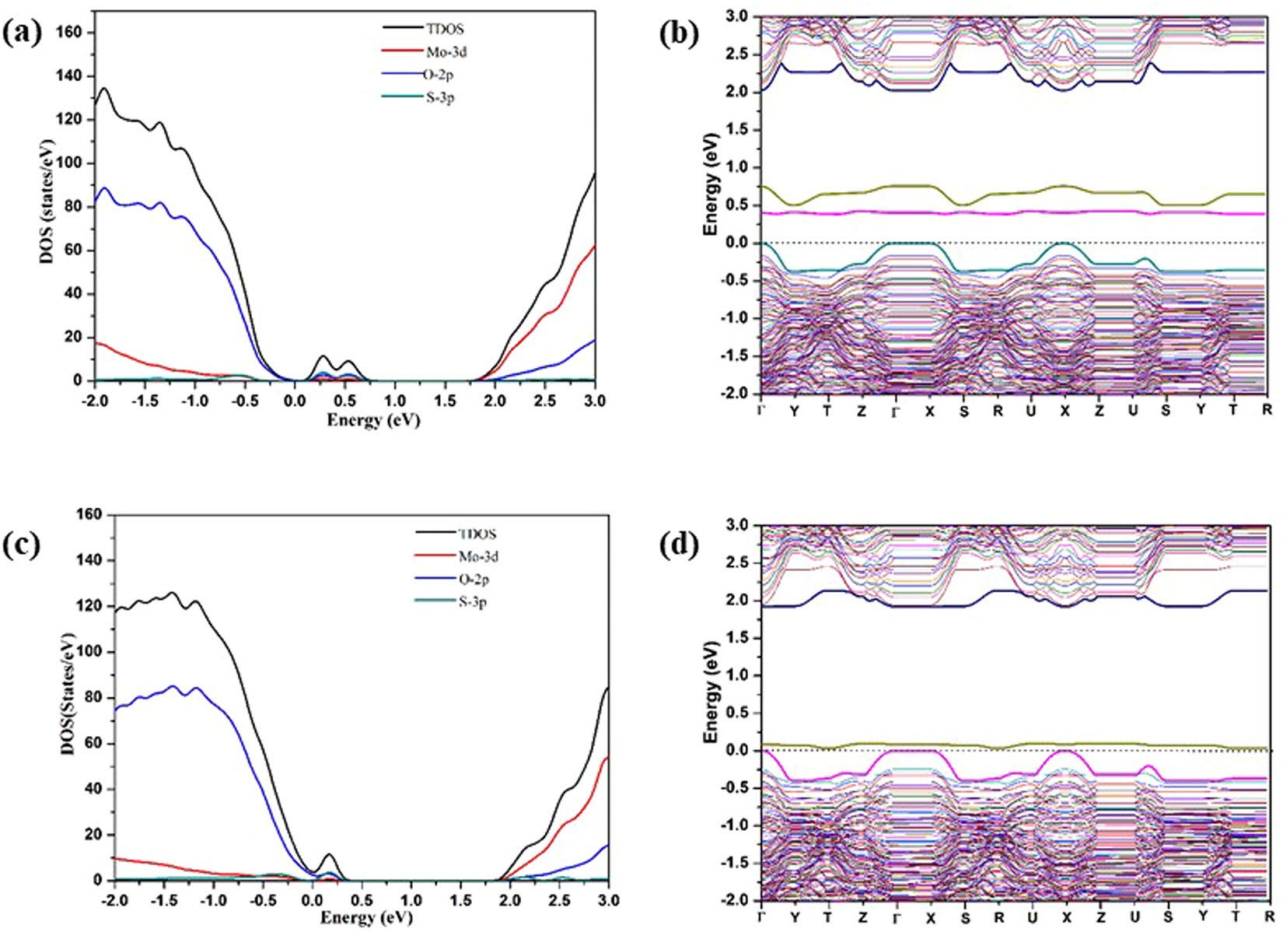
(**a**,**c**) The TDOS and the Mo 3d, O 2p and S 3p PDOS, (**b**,**d**) band structures of MoO_2.94_(S_t_S_a_)_0.06_ and MoO_2.91_(S_t_S_a_S_s_)_0.09_. Zero energy is chosen for the VBM.

The calculated densities of states and band structures of MoO_2.94_(S_t_S_a_)_0.03_ and MoO_2.91_(S_t_S_a_S_s_)_0.03_ are shown in Fig. 6. Two impurity bands are observed in the band gap for MoO_2.94_(S_t_S_a_)_0.03_, whereas only a single impurity band is found lying close to the VBM for MoO_2.91_(S_t_S_a_S_s_)_0.03_. The IBs of the MoO_2.94_(S_t_S_a_)_0.03_, as shown in PDOS, are essentially the superposition of the IBs of the S_t_ and S_a_ *mono-*doped structures, which is reasonable as the local geometries around the S_t_ and S_a_ dopants of the *bi-*doped structure are similar to the corresponding local geometries in the *mono-*doped structures. The higher energy IB for MoO_2.94_(S_t_S_a_)_0.03_ is mainly ascribed to the S 3p lone pair on S_a_, and the lower energy IB is the superposition of the S 3p lone pair on S_t_ and the 3p state of S_a_ that contributes to the longer Mo-S_a_ bond whose energy level is raised due to the John-Teller effects caused by two unequal Mo-S_a_ bonds (Fig. S4). The number of the IB decreases from two to one when an additional S dopant is added to the *bi-*doped MoO_2.94_(S_t_S_a_)_0.03_ structure to form local Mo surrounded by three dopant atoms in the resulting *tri*- S doped MoO_2.91_(S_t_S_a_S_s_)_0.03_ structure. This suggests that the IB of the *tri*-doped structure is no longer a simple superposition of the IBs of the *mono-* and *bi-* doped structures, in contrast to the cases with lower local dopant concentrations. This is due to the substantial changes of the local geometry and electronic structure upon the introduction of the third S dopant onto the same Mo site. The PDOS projected to the dopant atoms (Fig. S5 in the SI) shows that, the S_a_ dopant atoms only give rise to one IB in MoO_2.91_(S_t_S_a_S_s_)_0.03_. In MoO_2.91_(S_t_S_a_S_s_)_0.03_, S_a_ forms two Mo-S bonds and one S-S bond, so its valence p lone pair state must be involved in a donor-acceptor type bond, which gives rise to an IB. The energy level of this IB is expected to be comparable with the energy level of the IB of MoO_2.97_(S_s_)_0.03_ due to the similar bonding natures. Similarly, the over-coordinated S_s_ of MoO_2.91_(S_t_S_a_S_s_)_0.03_ will give rise to an IB at approximately the same energy level. We have learnt earlier that the IB of the MoO_2.97_(S_t_)_0.03_ is close in energy than the IB of MoO_2.97_(S_s_)_0.03_. Therefore, the S_a_, S_s_ and S_t_ of MoO_2.91_(S_t_S_a_S_s_)_0.03_ each give rise to an IB above the VBM, and the three IBs with similar peak positions overlap with each other to appear as one IB.

The band gap of MoO_2.94_(S_t_S_a_)_0.06_ is ~2.0 eV, slightly smaller than the band gaps of MoO_2.97_(S_a_)_0.03_ and MoO_2.97_(S_t_)_0.03_. A larger band gap reduction was observed for MoO_2.91_(S_t_S_a_S_s_)_0.09_, with the band gap calculated to be ~1.88 eV. The positions of the IB of MoO_2.91_(S_t_S_a_S_s_)_0.09_ is close to the VBM, and therefore the energy gap between the IB and CBM is larger than the IB-CBM gaps for the *mono*- and *bi-*S doped MoO_3_.

**Figure 7:**
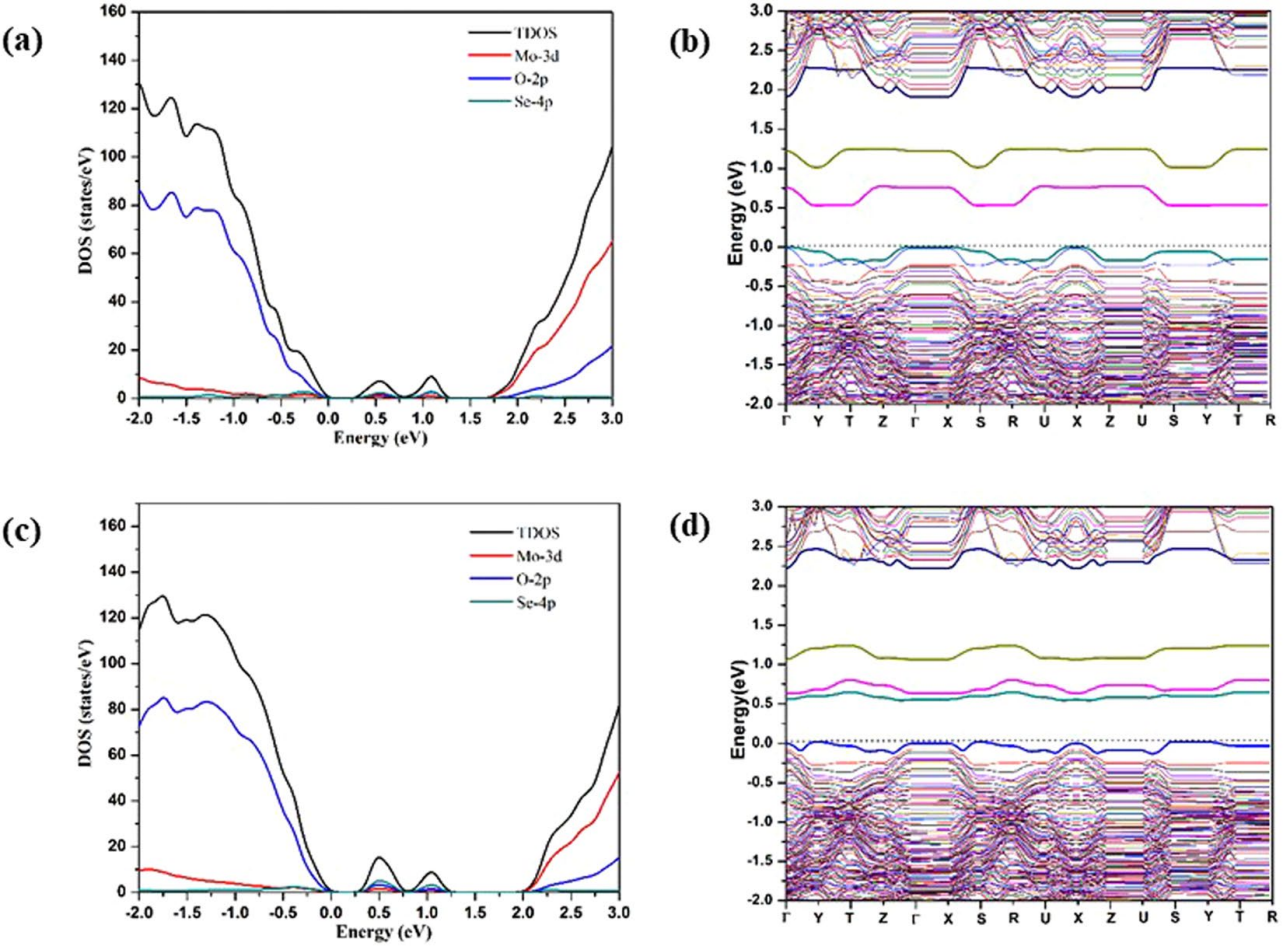
(**a**,**c**) are the TDOS and the Mo 3d, O 2p and S 3p PDOS, (**b**,**d**) are band structures of MoO_2.94_(Se_t_Se_a_)_0.03_ and MoO_2.91_(Se_t_Se_a_Se_s_)_0.03_ and the zero energy is chosen for the VBM.

For *bi*-/*tri*- Se doped structures MoO_2.94_(Se_t_Se_a_)_0.03_ and MoO_2.97_(Se_t_Se_a_Se_s_)_0.03_, optimized structures are shown in Fig. S1. In MoO_2.94_(Se_t_Se_a_)_0.03_, the Se dopants are essentially located at the original O sites. In MoO_2.97_(Se_t_Se_a_Se_s_)_0.03_, no Se_a_-Se_s_ bond is found, in contrast to the case of MoO_2.97_(S_t_S_a_S_s_)_0.03_ where a dopant-dopant bond is found; instead, a Se_a_-O_a_ bond is formed with a bond distance of 1.890 Å. In addition, the Se_s_ dopant is found to be out of the original O_s_ position into a new position on the Mo layer plane by rotating about the adjacent Mo-Mo axis (Fig. S1). The calculated DOS’s and band structures for MoO_2.94_(Se_t_Se_a_)_0.03_ and MoO_2.91_(Se_t_Se_a_Se_s_)_0.03_ are shown in Fig. 7. Two IBs were found for both MoO_2.94_(Se_t_Se_a_)_0.03_ and MoO_2.97_(Se_t_Se_a_Se_s_)_0.03_ (by omitting the new bands that are too close to the VBM and CBM), but the origins for the IBs of the two cases are different. For the MoO_2.94_(Se_t_Se_a_)_0.03_, the DOS of the IBs is approximately a superposition of the DOS’s of the IBs of MoO_2.94_(Se_t_)_0.03_ and MoO_2.94_(Se_a_)_0.03_. Such superposition is supposed to give rise to three IBs at 0.2, 0.5 and 0.8 eV above the VBM, but the lowest energy IB is too close to the VBM and thus appears to be absorbed therein. For MoO_2.97_(Se_t_Se_a_Se_s_)_0.03_ the composition of the IBs is totally different. The lower energy IB of the *tri-*Se doped structure is approximately equally contributed by the p states of Se_t_, Se_a_, and Se_s_. The higher energy IB is contributed by the p states of Se_a_ and Se_t_ (Fig. S4 in the SI). The distinct difference between the electronic structures of MoO_2.94_(Se_t_Se_a_)_0.03_ and MoO_2.97_(Se_t_Se_a_Se_s_)_0.03_ are apparently raised by the local geometry difference (Fig. S1(c,d); Table S4 in the SI) although the reason for the energy upshifts of the p states of Se_t_ from the *bi*-Se doped structure to the *tri*-Se doped structure is not clear, which is plausibly due to the stronger interactions between the Se dopants in the *tri*-S doped structure than in the *bi*-S doped structure. The band gaps of *bi*-Se and *tri*-Se doped systems are ~1.9 eV and ~2.25 eV respectively, both smaller than the band gap of the pure material. The maximum edges of the highest energy IBs of the *bi*-Se and *tri*-Se doped systems are much higher in energy than VBM, and the energy difference between the maximum edge of highest energy IB and the CBM is ~1 eV for MoO_2.94_(Se_t_Se_a_)_0.03_ and is 1.5 eV for MoO_2.97_(Se_t_Se_a_Se_s_)_0.03_. This suggests that MoO_2.94_(Se_t_Se_a_)_0.03_ may have the best absorption properties in the red and infrared regions among all the studied systems.

We have learnt that the O_t_ site is the most energetically favorable doping site for the *mono*-S and Se doping of MoO_3_. In real situation when the doping concentration is low, the newly introduced dopant tends to replace another O_t_ site rather than the O_a_ and O_s_ sites about the S_t_ (or Se_t_). Thus, the *bi*-S_t_ and *tri*-S_t_ doped systems with O_t_ being exclusively substituted were also calculated using the MoO_2.94_(S_t_)_0.06_ (Mo_32_O_94_S_2_) and MoO_2.91_(S_t_)_0.09_ (Mo_32_O_94_S_2_) models, to show the effects of the dopant concentration on the electronic structures, corresponding S/Se doped at different O_t_ position results are shown in the Figs S2 and S6 in the SI. According to the DOS diagrams (Fig. S6 in the SI), the same number of impurity bands are observed for both cases (S at same Mo-site and S doped at different O_t_ positions). The band gap remains the same as the doping concentration increases with the dopant atoms replacing the same type of O site. As expected, the populations of the impurity bands increase as the doping concentration increases, which leads to the high absorption strength for the peaks that are associated with the optical excitations involving the impurity bands (Fig. S7 in the SI).

### Optical proprieties of the doped MoO_3_

**Figure 8:**
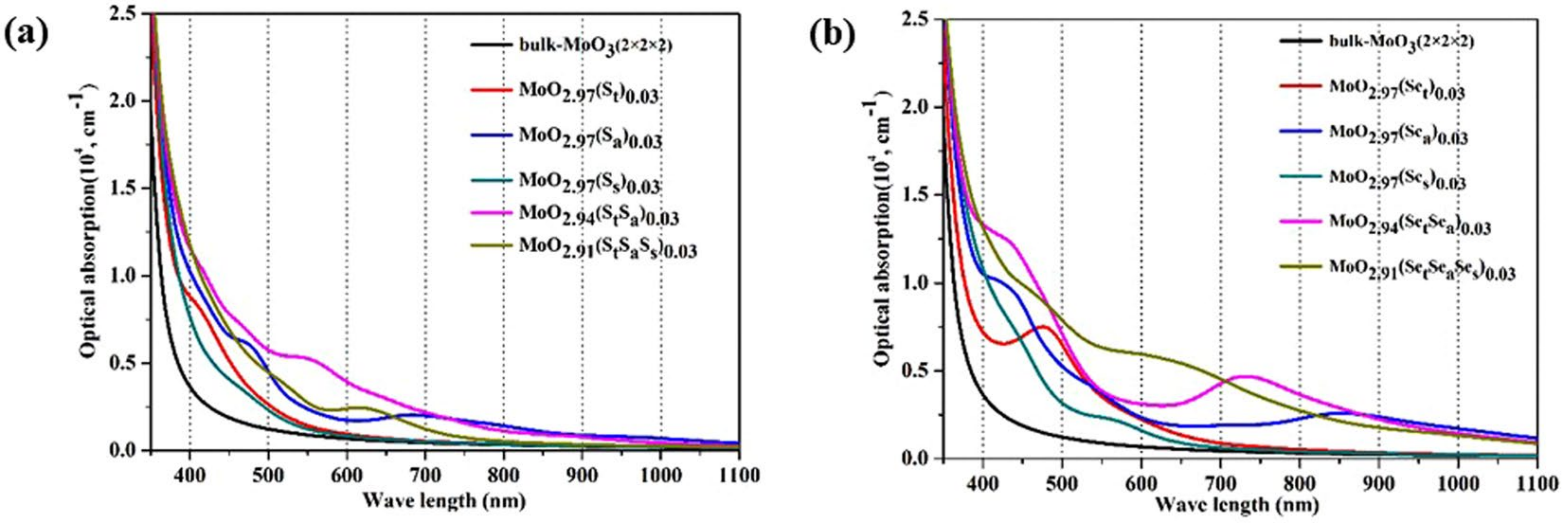
Calculated optical absorption spectra of **(a)** S-doped MoO_3_ and **(b)** Se-doped MoO_3_ using PBE+U method.

The optical absorption spectra for the pure and doped (S- and Se-) α-MoO_3_ systems were calculated, and the results are shown in Fig. 8. The optical absorption spectra of pure and doped systems are shown for wavelength >400 nm. The pure material has negligibly small absorption in this wavelength region. Notable increases in the absorption strength are found for the optical absorption spectra of the doped materials. All the S-doped structures in Fig. 8a have stronger absorption in the blue light region (450–495 nm), but only MoO_2.97_(S_a_)_0.03_, MoO_2.94_(S_t_S_a_)_0.03_ and MoO_2.91_(S_t_S_a_S_s_)_0.03_ exhibit slightly enhanced absorption in the red light (620–750 nm) and infrared regions. The optical absorption strengths for the locally *tri-*doped MoO_2.91_(S_t_S_a_S_s_)_0.03_ are overall weaker than those for the locally *bi-*doped MoO_2.94_(S_t_S_a_)_0.03_. This is because the change of the local geometry at high local dopant concentration lowers the energy of the IBs, and hence increases the gap between the maximum edge of the highest energy IB and CBM, which reduces the excitations in red and infrared regions for MoO_2.91_(S_t_S_a_S_s_)_0.03_. Se-doped systems show better absorption enhancements in the visible and infrared region than do the S-doped systems, in terms of the absorption strength increases as compared to absorption spectrum of the pure material. The Se-doped MoO_2.97_(Se_a_)_0.03_, MoO_2.94_(Se_t_Se_a_)_0.03_ and MoO_2.91_(Se_t_Se_a_Se_s_)_0.03_ structures also show good absorption strengths in the infrared region, which is not found for the S-doped structures. The superior optical properties (in the visible and infrared region) of the Se-doped systems are likely due to the higher energy levels of the valence p orbitals of Se than the p orbitals of S (that leads to higher energy maximum IB edges). Similar to the S-doping cases, the optical performance of S-doped increases as the local dopant concentration increases, until it reaches a point that the Mo sites of the material incorporate two Se dopants, as found in the MoO_2.94_(Se_t_Se_a_)_0.03_ structure model. Further increasing the doping concentration will generate Mo sites that incorporate over three Se dopant atoms, as found in MoO_2.91_(Se_t_Se_a_S_s_)_0.03_, which leads to a local geometry change that the dopants are displaced from the original O sites. As expected, for all the doped structures, the number of distinct absorption peaks in the infrared region are consistent with the number of distinct IBs between the VBM and CBM, since the excitations from each IB to conduction band levels near the CBM account for a new absorption peak in the optical spectra. The wavelength of the new adsorption peak can be approximately estimated as the energy difference between certain IB level and the center of the low-lying CB of the doped material. Overall, the optical absorption enhancements ascribed to the emergence of IBs were observed for the S and Se doped materials. The correlations between the new adsorption peaks and the dopant-induced IBs are analyzed and explained. Our results provide new insight for tuning the electronic and optical properties of MoO_3_.

## Conclusions

Using the first-principles calculations, we have studied the electronic structures and optical properties of sulfur and selenium doped α-MoO_3_. Doped structures with S or Se replacing several distinct O sites were looked into. From the formation energies, the S and Se prefer being doped into the O_t_ sites than the O_a_ and O_s_ sites. The reaction energies for doping α-MoO_3_ with solid-phase S and Se are 2.95 and 1.92 eV per dopant, respectively. Both doping reactions are found to be highly endothermic; the Se-doping reaction is more favorable than the S-doping reaction.

Significant changes were observed for the electronic structures as well as the optical absorption properties of α-MoO_3_ upon the S- and Se- doping treatments. The calculated band gap of the α-MoO_3−x_S_x_ slightly decreases as the doping concentration increases mostly due to the dopants levels that are closed to the CBM. For all of the doped structures, IBs were found between the VBM and the CBM. For MoO_2.97_(S_a_)_0.03_, MoO_2.94_(S_t_S_a_)_0.03,_ MoO_2.97_(Se_a_)_0.03_ and MoO_2.94_(Se_t_Se_a_)_0.03_, two distinct IBs were found. The energy levels and the number of the IBs greatly depend on the local molecular geometries and the local electronic structures. At low local doping concentration (number of S or Se < 3 about a Mo), in terms of number of dopant about a Mo center, the dopants will replace O atoms and stay on-site. As a consequence, the effects of the dopants in altering the electronic structures are additive. Hence, increasing the doping concentration at low concentration will enhance the optical absorption properties of the material in the visible and infrared regions. At high local doping concentration (number of S or Se ≥ 3 about a Mo), the net doping effect is no longer the superposition of the effects of the individual on-site dopant atoms as found for the low-concentration cases. The local geometries are greatly affected by the additional dopant atoms at high local doping concentration: dopant atoms are dislocated, Mo-S/Mo-Se bond distances are changed, and bonds between main group atoms, such as S-S and Se-O, are formed. Such local geometry changes turn out to substantially affect the band structures, especially to increase the gap between the maximum IB edge and CBM, which in turn applies a negative effect on the optical absorption properties in the visible and infrared regions. The optical absorption spectra were calculated for the studied systems. New absorption peaks with moderate intensities were found in the visible and infrared regions for the doped structure, especially for MoO_2.94_(S_t_S_a_)_0.03_ and MoO_2.94_(Se_t_Se_a_)_0.03_; such absorption peaks are beneficial for solar energy harvesting. Correlations between the IBs and the new absorption peaks in the visible and infrared region were found. Based on our first-principles calculation results, the designed S- and Se-doping at different concentrations appear to be effective for tuning of the band gaps and optical properties of MoO_3_. Our study suggests that the isovalent doped-MoO_3_ with economically viable dopants may be utilized in water oxidation and many other photochemical applications, although further computational and experimental efforts are required for more thorough investigations.

## Electronic supplementary material

Supplementary Information
