# Supplementary material for: Tweaking the Electronic and Optical Properties of α-MoO3 by Sulphur and Selenium Doping – a Density Functional Theory Study
Source: Sci Rep. 2018 Jul 4;8:10144. doi: 10.1038/s41598-018-28522-7 (PMC6031609; doi:10.1038/s41598-018-28522-7)
Supplement: Supplementary file 1 — Supplementary Information [file 41598_2018_28522_MOESM1_ESM.pdf]

Supplementary Information

Tweaking the Electronic and Optical Properties of α-MoO_3 by Sulphur and Selenium Doping – a Density Functional Theory Study

Sateesh Bandaru[1], Govindarajan Saranya[1], Niall J English[2], Chiyung Yam,[1]* Mingyang Chen[1]*

[1]Beijing Computational Science Research Center, Beijing 100084, China
[2]School of Chemical and Bioprocess Engineering, University College Dublin, Belfield, Dublin 4, Ireland.

Corresponding author: yamcy@csrc.ac.cn; mychen@csrc.ac.cn

Table S1: Lattice parameters and Mo-O_t, Mo-O_s, and Mo-O_a, band gap energies (are in eV) bond distances (are in Å) have computed using different U_{eff} values and corresponding experimental values are shown.

		U_{eff}						
Lattice parameter	*Exptl.*[a]	4.38	5.0	6.0	8.0	8.2	8.6	10.2
a(Å)	3.96	3.96	3.96	3.96	3.96	3.96	3.96	3.96
b(Å)	13.86	13.85	13.85	13.85	13.85	13.85	13.85	13.85
c(Å)	3.69	3.69	3.69	3.69	3.69	3.69	3.69	3.69
Band gap (eV)		2.13	1.55	2.27	2.47	2.17	2.58	2.32
Bond lengths								
Mo-O_t (Å)	1.67	1.69	1.70	1.69	1.68	1.70	1.68	1.70
Mo-O_s (Å)	1.95	1.95	1.94	1.95	1.95	1.94	1.95	1.94
Mo-O_a (Å)	1.73,	1.77	1.77	1.78	1.78	1.78	1.78	1.78
	2.25	2.21	2.21	2.20	2.20	2.21	2.20	2.20

[a]: L. Kihlborg, *Ark. Kemi*, 1963, 21, 357

Table S2. Energy gap between the impurity band and VBM for $MoO_{2.97}(S_t)_{0.03}$ calculated using different U_{eff} values.

U_{eff} in eV	IB-VB in eV
4.8	0.24
5.0	0.25
6.0	0.25
8.0	0.26
8.2	0.25
8.6	0.26

Table S3: Mo-O and Mo-S bond distances (in Å) of bulk-MoO_3(2x2x2) and all doped structures. [a]

	$Mo\text{-}O_t$	$Mo\text{-}O_a$	$Mo\text{-}O_s$	$Mo\text{-}S_t$	$Mo\text{-}S_a$	$Mo\text{-}S_s$	Other
Bulk MoO3 (2x2x2)	1.684	2.205<br>1.778	1.951<br>2.381[b]				
$MoO_{2.97}(S_t)_{0.03}$	-*a*-	2.222<br>1.777	1.957	2.073			
$MoO_{2.97}(S_a)_{0.03}$	1.679	-*a*-	1.942		2.333<br>2.166		
$MoO_{2.97}(S_s)_{0.03}$	1.704	2.305<br>1.774	-*a*-			2.297<br>2.712[b]	
$MoO_{2.94}(S_tS_a)_{0.03}$	-*a*-	-*a*-	1.950	2.060	2.317<br>2.160		
$MoO_{2.91}(S_tS_aS_s)_{0.03}$	-*a*-	-*a*-	-*a*-	2.068	2.391<br>2.357	2.352<br>2.479<br>2.712[b]	$S_a\text{-}S_s$:<br>2.045

[a] -*a*- represents the S and Se-doping positions

[b] interlayer bond distances

Table S4: Mo-O and Mo-Se bond distances (in Å) of bulk-MoO_3(2x2x2) and all doped structures. [a]

	Mo-O_t	Mo-O_a	Mo-O_s	Mo-Se_t	Mo-Se_a	Mo-Se_s	Other
Bulk MoO3 (2x2x2)	1.684	2.205<br>1.778	1.951				
$MoO_{2.97}(Se_t)_{0.03}$	*-a-*	2.227<br>1.778	1.962	2.199			
$MoO_{2.97}(Se_a)_{0.03}$	1.679	*-a-*	1.944		2.445<br>2.270		
$MoO_{2.97}(Se_s)_{0.03}$	1.690	2.357<br>1.795	*-a-*			2.405<br>2.842[b]	
$MoO_{2.94}(Se_tSe_a)_{0.03}$	*-a-*	*-a-*	1.963	2.185	2.450<br>2.275		
$MoO_{2.91}(Se_tSe_aSe_s)_{0.03}$	*-a-*	*-a-*	*-a-*	2.224	2.627<br>2.563	2.433<br>2.470<br>3.046[b]	Se_a-Se_s:<br>3.117<br>Se_a-O_a:<br>1.890

[a] *-a-* represents the S and Se-doping positions

[b] interlayer bond distances

Reaction energy for the doping reaction of MoO_3 with solid S dopant.

$$O_t + S(solid) \rightarrow S_t + \frac{1}{2}O_2(gas)$$

$$E_{rxn}(real) = E_S + \frac{1}{2}O_2 - E_O - \frac{1}{\infty}S(bulk)$$

$$E_2 = E_{form} - \Delta H_f(O) + \Delta H_f(S)$$

Table S5: Formation energies (in eV) and reaction energies (in eV) of *mono-*, *bi-* and *tri-* S and Se doped MoO_3 systems.

Structure	E_{form}	E_{rxn}		E_{form}	E_{rxn}
$MoO_{2.97}(S_t)_{0.03}$	2.64	2.95	$MoO_{2.97}(Se_t)_{0.03}$	2.15	1.92
$MoO_{2.97}(S_a)_{0.03}$	3.78	4.09	$MoO_{2.97}(Se_a)_{0.03}$	3.76	3.53
$MoO_{2.97}(S_s)_{0.03}$	3.63	3.94	$MoO_{2.97}(Se_s)_{0.03}$	3.45	3.22
$MoO_{2.94}(S_tS_a)_{0.03}$	6.34	6.96	$MoO_{2.94}(Se_tSe_a)_{0.03}$	5.82	5.36
$MoO_{2.91}(S_tS_aS_s)_{0.03}$	6.85	7.78	$MoO_{2.91}(Se_tSe_aSe_s)_{0.03}$	6.97	6.28

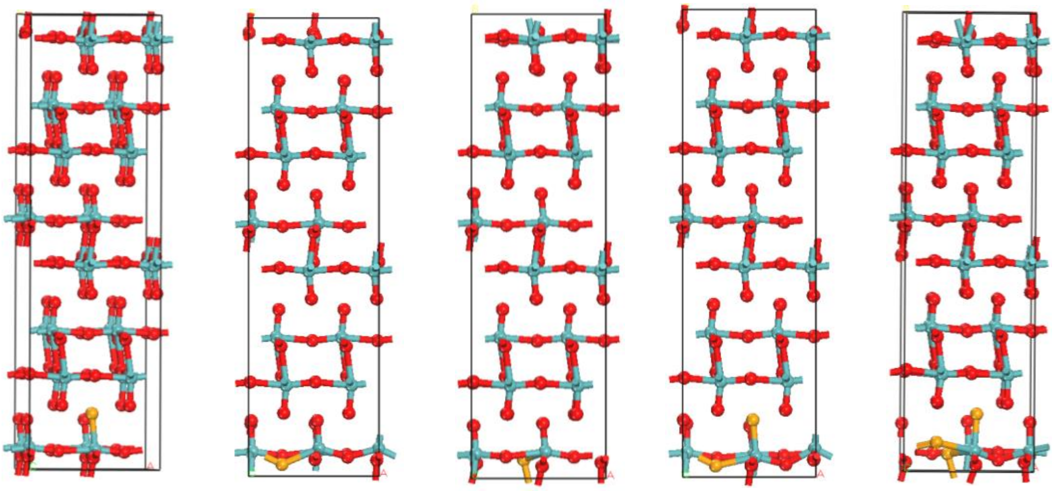

(a) (b) (c) (d) (e)

Figure S1: Constructed supercell for the (a) (b) and (c) are the *mono*-Se doped $MoO_{2.97}(Se_t)_{0.03}$, $MoO_{2.97}(Se_a)_{0.03}$, and $MoO_{2.97}(Se_s)_{0.03}$ respectively; (d) is $MoO_{2.94}(Se_tSe_a)_{0.03}$ is *bi*-Se and (e) is *tri*-S $MoO_{2.91}(Se_tSe_aSe_s)_{0.03}$ in both these cases Se atoms are doped at same Mo-site in the MoO_3 (2×2×2) super cell.

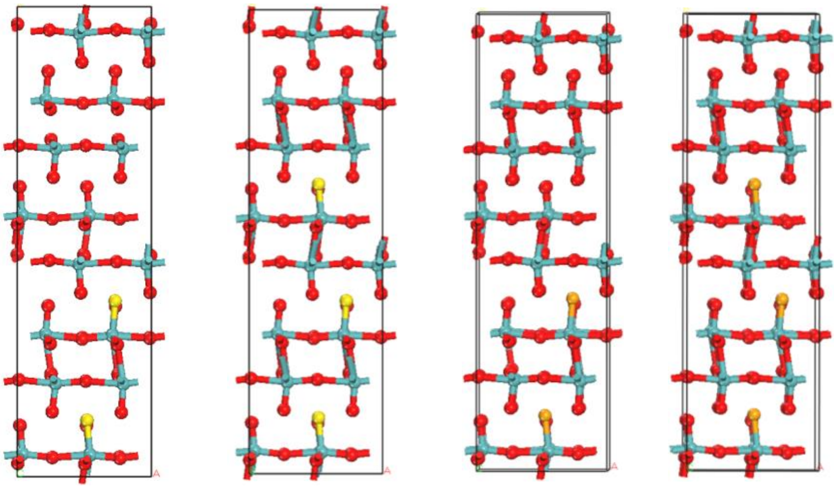

(a) (b) (c) (d)

Figure S2: Constructed supercell for the (a) *bi*-S doped $MoO_{2.94}(S_t)_{0.06}$ and (b) the *tri*-S doped $MoO_{2.91}(S_t)_{0.09}$ (c) *bi*-Se doped $MoO_{2.94}(Se_t)_{0.06}$ and (d) the *tri*-Se doped $MoO_{2.91}(Se_t)_{0.09}$.

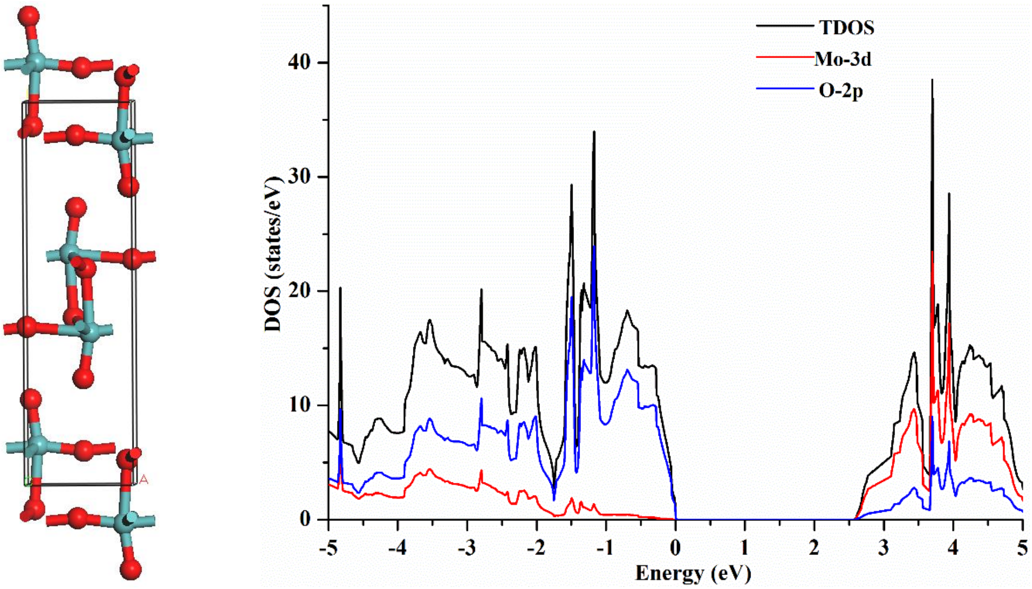

(a) (b)

Figure S3: (a) Bulk-MoO_3 (b) Density of states of unit cell of bulk MoO_3 structure at the PBE+U level.

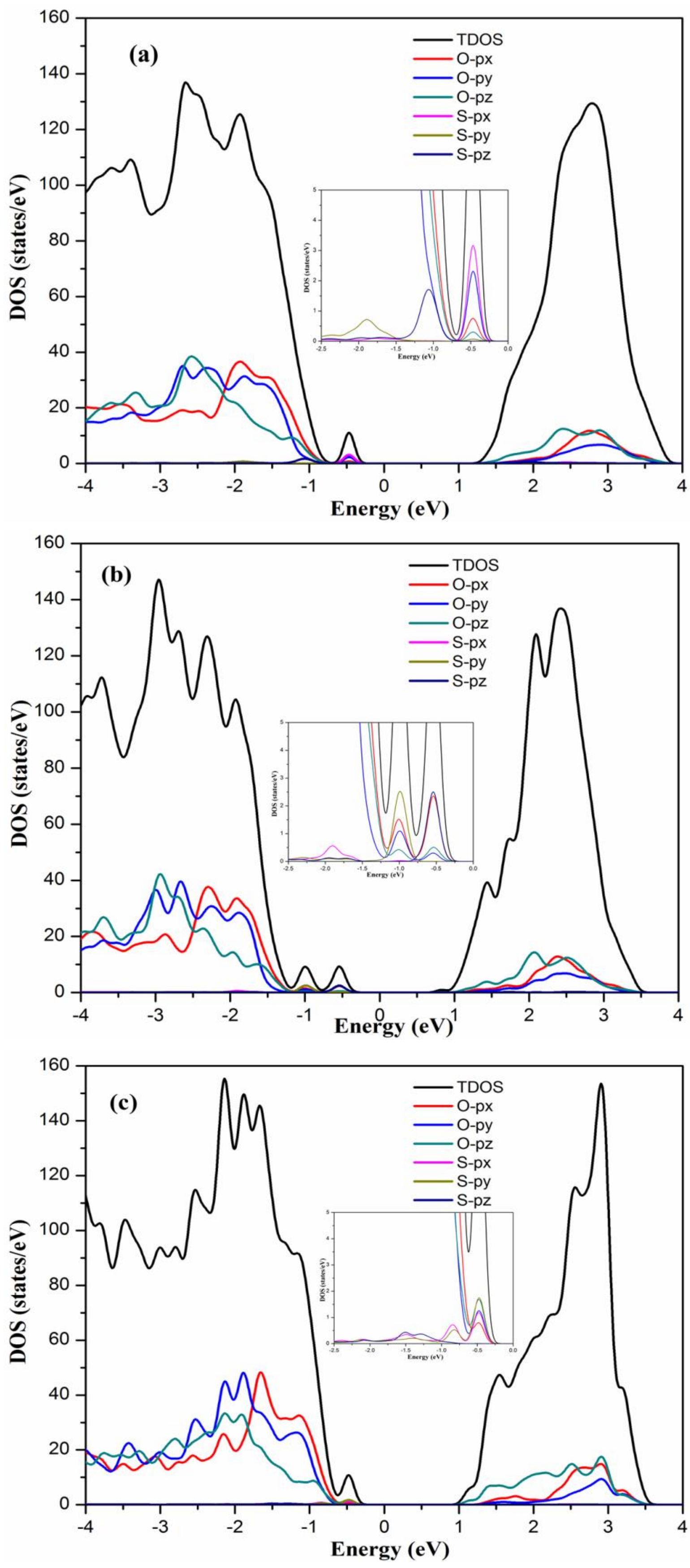

Figure S4: TDOS and PDOS on the p orbitals of O and S in (a) $MoO_{2.97}(S_t)_{0.03}$, (b) $MoO_{2.97}(S_a)_{0.03}$ and (c) $MoO_{2.97}(S_s)_{0.03}$. The Fermi level is set zero.

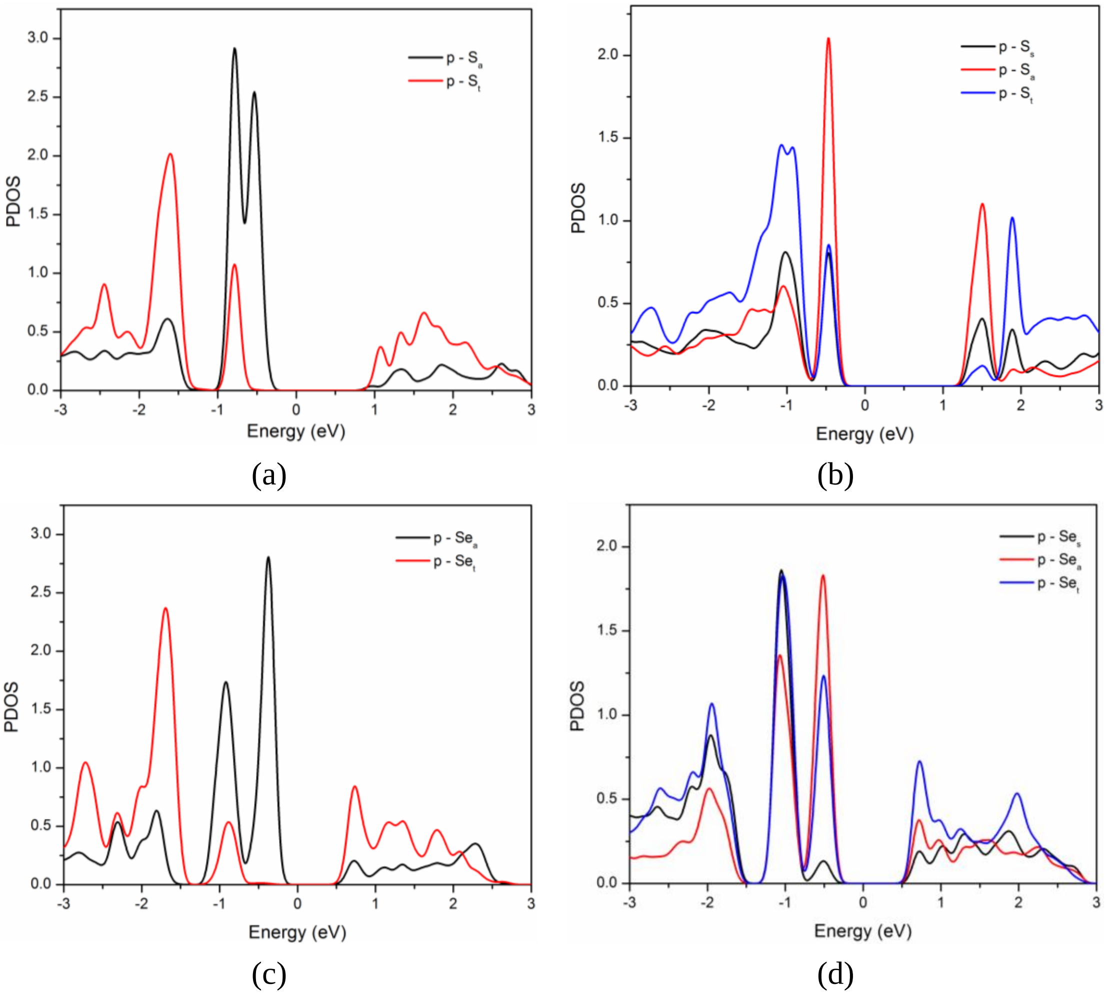

Figure S5: (a) and (b) are PDOS of the S dopant atoms in the $MoO_{2.94}(S_tS_a)_{0.03}$ and $MoO_{2.91}(S_tS_aS_s)_{0.03}$; (c) and (d) are PDOS of the Se dopant atoms in the $MoO_{2.94}(Se_tSe_a)_{0.03}$ and $MoO_{2.91}(Se_tSe_aSe_s)_{0.03}$ using the PBE+U functional.

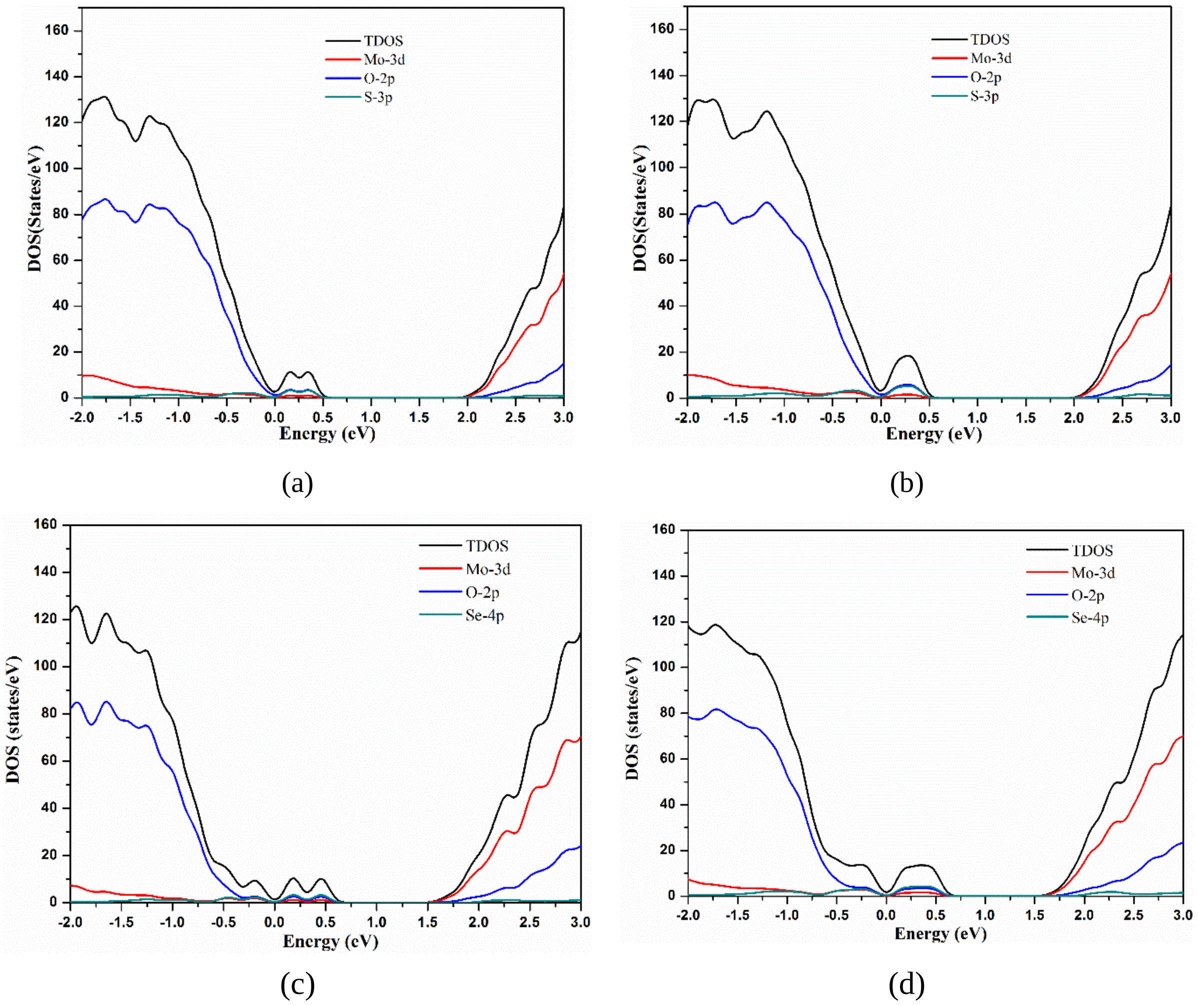

Figure S6: (a) and (b) are the total density of states (TDOS) and the Mo 3d, O 2p and S 3p projected density of states of (a) $MoO_{2.94}(S_t)_{0.06}$, (b) $MoO_{2.91}(S_t)_{0.09}$ and (c) $MoO_{2.94}(Se_t)_{0.06}$, (d) $MoO_{2.91}(Se_t)_{0.09}$ and the zero energy is chosen for the VBM.

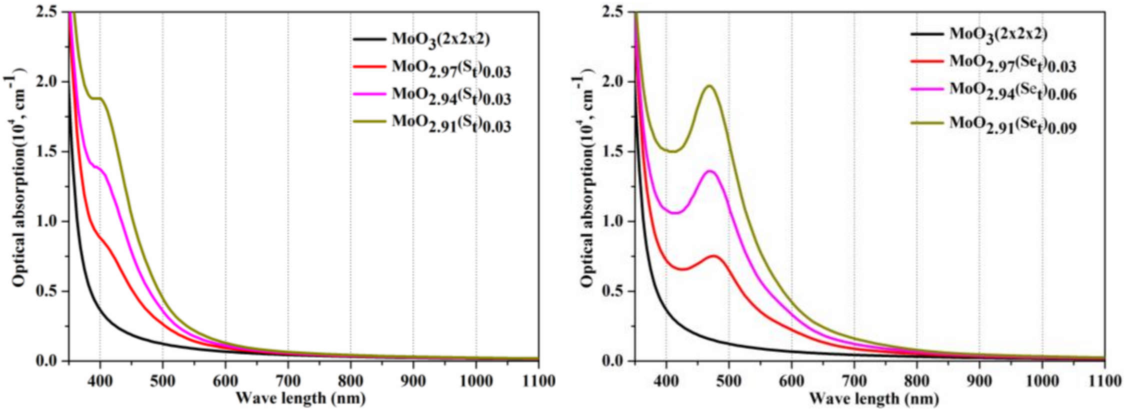

(a) (b)

Figure S7: Calculated optical absorption spectra of pure bulk MoO_3 (2×2×2) and (a) S doped at the O_t positions in MoO_3(2x2x2) with the increasing dopant concentration and (b) Se doped at the O_t positions in MoO_3(2x2x2) with the increasing dopant concentration using PBE+U method.
